# Clinical Cases

**Published:** 1893-02

**Authors:** B. Fincke

**Affiliations:** Brooklyn, N. Y.


### CLINICAL CASES.

B. Fincke, M.D., Brooklyn, N. Y.

1. Mrs. D. June 26th, 1859. 3 P. M.—Chill last night at 11 P. M. with pains in all the bones and slight thirst, lasting one hour and a half; then fever, lasting till morning with perspiration. Vomiting to-day. Pain in passing water. Constipation, for which she took Castor Oil with immediate effect; also gin and water without effect. ℞. Pulsatilla-nig. 5m (F.) three pellets. Cured.

2. Mrs. D. June 11th, 1864.—Felon on the right thumb for one week. Cut this morning in several places, looking frightful, suppurating, with violent sticking pains all along the arm. The physician says she will lose her bone. The cutting made everything worse. ℞. Silicea 40m (F.); a few pellets in half a tumbler of water; a teaspoonful every three hours. Seven powders.

June 15th.—The pains had subsided the second day. A large red blister formed at the back of the thumb below the nail; the thumb was in good shape and looked like healing. The woman coming five miles distance was astonished at the result and got well without anything else than the internal medication.

3. Mrs. D. Forty years; pregnant. February 28th, 1867. Echo of her own words, and of her own breathing; buzzing in her right ear after 4 P. M. ℞. Causticum cm (F.). Had an astringent taste and cured.

4. Mrs. S. January 3d, 1865. 11.15 A. M.—Since a fortnight toothache, slowly increasing till about five days ago, when the pains became very violent. Tearing and stinging and burning pains in the lower left three molars, later going into ears and forehead; incessant; ameliorated by cold water held in the mouth, and by external warmth. Tearing in the left leg below the knee downward. Sensation between the shoulders as of ice. Chilliness, thirst. Three days ago the lower jaw began to swell. Took yesterday Antimon. crud. without effect. She went to a dentist to have one of the affected molars drawn, which felt three times larger and had a flat cavity just above the gum, but the dentist refused because it would make her much worse. The woman declared afterward that she had a good deal of toothache in her life, but nothing like this before. She heated a knitting needle red hot and burned the tooth, but the help it afforded did not last long. Quarter past eleven o'clock A. M. ℞. Silicea cm (F.) two pellets.

January 6th.—The toothache was better immediately and decreased gradually. The tooth feels a little larger yet, but is not elongated. Pains almost all gone. Throbbing in the lower jaw in the bone below. The day when she took the medicine, between 3 and 4 P. M., violent tearing in the left cheek and left ear, which felt as if ulcerated, and did not bear the least touch. Yesterday terrible tearing pain in the left arm, mostly upper arm, with numbness of the fingers. After this ceased it was as if fire was running through with burning of the skin. Several times a stitch below the right short ribs going half-way through the body inward on a place where it was so painful yesterday, as from ulceration, that she could not bear touching it. Yesterday sensation in the splenic region inside like ice, then turning into violent heat for half an hour. She wakes up about 1 A. M. and cannot sleep any more. She took nothing else and was well.

5. Mrs. S. May 23d, 1865.—Whilst washing, standing on cold stones, on the third day of menstruation, this stopped, and, suddenly, bearing down of the womb came on with terrible hot cutting pains in the hypogastrium, more in the right side, going down the thigh like wild labor pains and around the navel. Half-past nine P. M. ℞. Pulsatilla-nig. 5m (F.).

After half an hour better. The pains went back very much as they came like in emptying a bottle, then heat and burning in the stomach, going up to œsophagus and out of the mouth. The places of pain are now sore as from a wound.

June 10th.—Menses did not return, but she was well.

6. George L——, merchant, August 9th, 1864, lived in Gowanus last May, got chills and fever, and was treated with Quinine. Chill came on at 10 A. M. and lasted till noon, followed by perspiration, with thirst through all the stages. It came on four or five times as tertiary, then it stopped. Took Quinine, seven grains at once, then five grains three times every day for eight days. Then the fever was gone.

But since then he gets the fever sometimes in the forenoon. Several days preceding it, tired pains in sacrum and lower extremities. Yesterday tired, languid, white fingers. Chill for one hour, shaking, some headache, nausea, then heat for fifteen minutes, then sweat for three hours. Urine dark with dark red sediment. Stool normal. After having had the fever a second time, swollen feet and eruption around lips and nose for eight days. Some yellowness of conjunctiva. Tongue rose-colored with enlarged papillæ, but when he has the fever the tongue is coated whitish-yellow. Sometimes gets an attack in one, two or five weeks, and he takes Quinine between times. Sleeps well, has an inborn aversion to wine, beer or other spirituous beverages, but loves coffee and tea passionately. Smokes a good deal. Had an attack of cholera five years ago treated allopathically by Opium. 9 A. M., ℞. Natrum-mur. 5m (F.). Several years after I learned from a patient sent by him that this dose cured him right up.

7. F. M——, a music teacher, short, thick-set, March 5th, 1874.—Had given a lesson in a heated room, gets a cold perspiration, nausea, a dreadful colic, the cold sweat running in streams all over his body, and he thinks he must die. 4 P. M., ℞. Arsenicum-a. 90m (F.). In ten minutes it was all gone and he was well.

8. Dr. B——, July 22d, 1867.—Sciatica in left lower limb, worse on coughing, laughing, sneezing, going to stool, straightening the limb, stepping lower than imagined. Rheumatism in the small of back preceded it. The pain goes from the foramen sciaticum down to the foot, which is numb. Suffers since June 28th. ℞. Tellur-met. 90m (F.). May 24th, 1874.—Helped him and since then he cures all the sciaticas of his patients with Tellur. 90m. So he told me to-day.

9. E. K.——, nineteen years, dark, florid complexion, tall and thin. October 12th, 1866, quarter of three P. M., was brought to my office by the late apothecary, Mr. Smith, bleeding from the nose profusely. I gave him at once a dose of Acon. cm (F.), which stopped the bleeding in a few minutes while in the office, and he did not bleed for two months after, when a little blood came from the right nostril.

This case was a fearful one. He was taken in Nassau Street, New York, bleeding all the time. When arriving at Smith's store the blood was streaming from both nostrils and filled a basin full. Mr. Smith held a piece of ice six inches square on the back of the neck, but it was not of the least avail. The bleeding continued all the way and here, till Acon. cm stopped it. That boy must have had a greater amount of blood in him than common. And yet he did not show much weakness, for he walked home again in a short while. He had valvular disease of the heart for which I treated him. For half a year he did not come near me because he felt so well. But then he struck his nose and bled for five minutes, when it stopped by itself. Later on, when I treated him for his heart disease, he bled again for an hour after over-exertion in warm weather, and that was the last I saw of him. He went from me to Dr. Boskowitz, and from him to an allopathic physician, under whose care he died a couple of years later, having got married in the meantime.

10. Mr. G., forty-five years, slight frame, merchant, July 22d, 1867. Since July 4th, from ice-cream and new potatoes, sinking in stomach. Bad taste in mouth. Heavy weight two and a half inches below the liver in the axillary line—"the hand can grasp it"—Dr. Willard Parker who examined him said it was not the liver—steady dull though not constant pain eased by hot cloths. Appetite tolerable. Qualmish, nauseous. Fried oysters, raw chestnuts disagree with him. Offensive breath. Sleeps badly. Confused dreams. When waking up, swelling of penis. Tongue slightly coated. Nasal catarrh, matter forming in the nose. No sense of smell. From any little cause, cold, or indigestion all the symptoms re-appear. Hands dry. Feet cold. Want of vital heat. Active exercise weakens him too much. Low-spirited, feels like crying. ℞. Nux-vom. 50m (F.).

Got well shortly and ever since remained well.

11. Mrs. W., fifty-odd years, February 15th, 1872.—Had severe pain in the pelvic region which made it difficult for her to walk, and rubbed on a mixture of Aconite tincture and Chloroform, though she ought to have known better, because on a former occasion she had used Chloroform and did not get over its effects for three to four years.

Now she is in an agony of pain, shrieking. Dr. R., a former homœopath, had diagnosed it a severe attack of hysteria, and gave Asafœtida. He said she could have smeared Aconite tincture all over her body and it would not have affected her in the least, which the old gentleman doubted, for he had seen a little Aconite tincture put over the eyelid, making the person immediately blind. ℞. Aconit.-nap. 3cm, in water, a teaspoonful every half-hour.

She was easier after an hour, and slept through the afternoon.

12. Thomas H., an Irish nursling, six months. August 29th, 1854.—Suffers for the last two months from vomiting and diarrhœa, since eleven days much worse. In this hot and dry weather patient was emaciated to a skeleton. Hands and feet cold. Incessant vomiting of greenish water and the milk taken. Yellow watery diarrhœa. Abdomen hard. Shrill crying. Convulsive movements of hands and feet. Sleep with half-open eyes. Boring the head into the pillow, hands reaching toward the back of the head. The child had convulsions two months ago, and has no tooth yet.

℞. Verat-a. 2400 (F.), one pellet at once to the child's tongue.

℞. Verat-a. 12 (F.), for the mother to be taken in half a tumbler of water, one teaspoonful every three hours.

August 31st.—The child is reported to be well. It did not get sick till next summer, when a slight attack was easily disposed of.

---

